# Complement regulator CD46: genetic variants and disease associations

**DOI:** 10.1186/s40246-015-0029-z

**Published:** 2015-06-10

**Authors:** M. Kathryn Liszewski, John P. Atkinson

**Affiliations:** Division of Rheumatology, Department of Medicine, Washington University School of Medicine, 660 South Euclid, Saint Louis, MO 63110 USA

**Keywords:** CD46, Membrane cofactor protein, Complement, Complement regulation, Atypical hemolytic uremic syndrome

## Abstract

Membrane cofactor protein (MCP; CD46) is an ubiquitously expressed complement regulatory protein that protects host cells from injury by complement. This type-I membrane glycoprotein serves as a cofactor for the serine protease factor I to mediate inactivation of C3b and C4b deposited on host cells. More than 60 disease-associated mutations in *MCP* have now been identified. The majority of the mutations are linked to a rare thrombotic microangiopathic-based disease, atypical hemolytic uremic syndrome (aHUS), but new putative links to systemic lupus erythematosus, glomerulonephritis, and pregnancy-related disorders among others have also been identified. This review summarizes our current knowledge of disease-associated mutations in this complement inhibitor.

## Introduction

The complement system is one of the most ancient components of innate immunity. It likely evolved from a C3-like protein that was cleaved by proteases into biologically active self-defense fragments to counteract invading microbes, particularly bacteria, and to clear biologic debris (self and foreign) [1, 2]. The development of a circulatory system may have been the key evolutionary pressure that drove the need for a rapidly acting (within seconds) innate immune host-defense process with the destructive power to prevent invasion and multiplication of bacteria in the blood stream [3]. The complement system is sometimes referred to as “the guardian of the intravascular space”.

**Fig 1:**
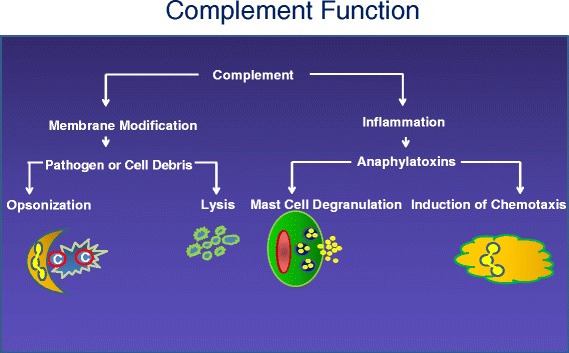
Complement function. The two primary functions of the complement system are to modify pathogens and self-debris with clusters of complement fragments (opsonization). This, in turn, facilitates interaction with complement receptors and, in some bacteria and viruses, induces lysis. The second function is to promote the inflammatory response. Complement fragments C3a and C5a generated during activation of the cascades stimulate many cell types. In the case of mast cells, release of immunomodulatory granules also attracts phagocytic cells into the area of inflammation (chemotaxis)

The vertebrate-complement system consists of a set of sequentially interacting proteins featuring three major pathways that provide a swift and powerful host-defense system. It promotes the inflammatory response and mediates the identification and destruction of pathogens. This is accomplished in two major ways (Fig. 1). First, complement modifies the membranes of microbes. Activated fragments are covalently deposited in large amounts on microorganisms, immune complexes, and damaged tissue. For example, several million C3-derived activation fragments can attach in clusters to a bacterial surface in less than five minutes. The most important function of these covalently deposited complement proteins is to opsonize the target, thus serving as ligands for complement receptors on peripheral blood cells; specifically, erythrocytes, neutrophils, B lymphocytes and monocytes, as well as dendritic cells, and tissue monocytes. The receptors serve to bind (immune adherence), internalize (in some cases), transport, and clear the microbe. Additionally, the membranes of some pathogens (especially gram-negative bacteria) can be disrupted by the terminal-complement components (membrane-attack complex, MAC) leading to osmotic lysis.

The second function of complement is to promote the inflammatory response. Thus, peptides, released by proteolysis during complement activation, bind to receptors to elicit an inflammatory reaction. These peptides are termed anaphylatoxins because they can trigger the release of mediators such as histamine to cause shock.

Due to its proinflammatory and destructive capabilities, it is no surprise that nearly half of the complement proteins serve in its regulation. Unimpeded, the complement system fires to exhaustion, a point illustrated by inherited deficiencies of its regulatory proteins [4, 5]. These inhibitors also participate in “self” versus “nonself” discrimination in that foreign surfaces, usually *lacking* such regulators, are recognized and attacked, while healthy self-tissues expressing the regulators are protected.

The alternative pathway (AP) continuously turns over, generating C3 fragments. If a C3b lands on host cells, it must be inactivated by regulatory proteins. One such control protein, CD46 (membrane cofactor protein; MCP), is a member of a group of genetically-, structurally-, and functionally-related proteins called the regulators of complement activation (RCA) [6, 7]. As its name implies, the goal of this gene cluster of receptors and inhibitors is to provide homeostasis by tightly controlling the rapid and powerful amplification process of the AP in order to focus complement attack, in both time and space, on pathogens and, in a more homeostatic manner, injured tissue.

Recently identified associations of human disease featuring excessive AP activation with heterozygous mutations in its components and regulators and the development of a novel therapeutic agent to block C5 cleavage have reignited interest in the field [8, 9].

This review focuses on the ubiquitously expressed inhibitor of C3b and C4b, CD46, and the primary diseases associated with its dysfunction. Citations included are not meant to be exhaustive, but rather to provide key review articles.

### Complement pathways

More than a billion years ago, primitive elements of the complement system arose to form a humoral immune system likely derived by proteolysis of a primeval protein whose fragmentation released one piece to mediate opsonization and a second one to elicit an inflammatory response [1, 10–12]. This original pathway (that remains today with enhanced sophistication and inappropriately called the AP) provided a simple protein-based recognition and effector scheme against pathogens. Lectins and antibodies, representing subsequent evolutionary developments, became connected to the complement-dependent effector mechanisms of opsonization and membrane perturbation. As the system grew in capacity and efficiency, control mechanisms were required to maintain homeostasis and to focus attack on pathogens while minimizing damage to self.

**Fig. 2:**
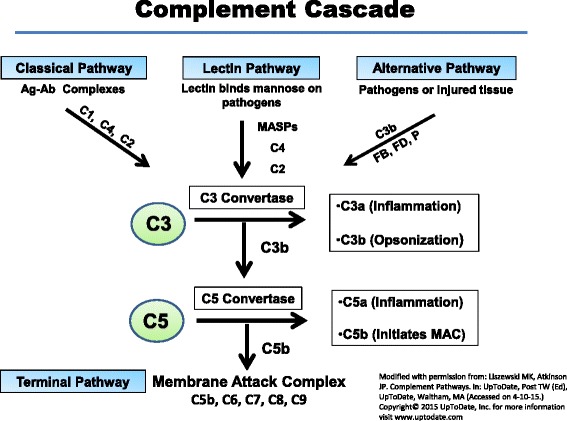
The complement cascades. The three pathways of complement activation are shown. Although each is triggered independently, they merge at the step of C3 activation. The CP is initiated by the binding of antibody to antigen and the lectin pathway by the binding of lectin to a sugar. The alternative pathway turns over continuously and possesses a feedback loop (see Fig. 3). Activation of the complement system leads to inflammation, opsonization, and membrane perturbation. *Abbreviations*: *MASP* MBL-associated serine protease, *MBL* mannose-binding lectin, *FB* factor B, *FD* factor D, *P* properdin

The contemporary human-complement system now consists of an efficient, interacting set of nearly 60 blood (serum) and cellular components that include components of the activating cascades, receptors, and positive and negative regulators. Complement systems similar to that in mammals also have been identified in birds, fish, amphibians, and reptiles. An AP is also found in more primitive species, even those lacking a circulatory system [13]. The complement system consists of three major activating pathways that are independently triggered, yet all have the common goal of modifying the target membrane by depositing C3 activation products and then engaging a common terminal membrane-attack complex (Fig. 2).

**Fig. 3:**
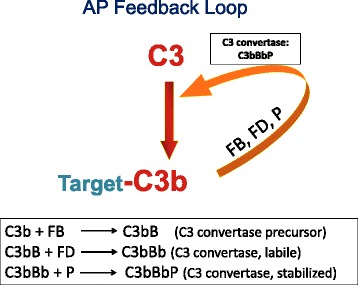
Feedback loop of the alternative pathway. Following the attachment of C3b to its target, a feedback loop can be engaged via interactions with the two proteases, factor B (FB) and factor D (FD), to form the AP C3 convertase. The binding of properdin (P) stabilizes the complex (i.e., its half-life is increased from 30–40 s to 3–4 min). Within a few minutes, more than one million C3bs can be generated and bound to a single bacterium

The AP is the most ancient cascade. It does not require an antibody, a lectin, or prior contact with a pathogen to become engaged. Indeed, it serves as a rapid, self-amplifying, and exceptionally powerful innate immune system capable of independently recognizing and destroying foreign targets and promoting an inflammatory response. A small amount of auto-activated C3 (so called, C3 tickover) is constantly generated in blood secondary to engagement of its labile thioester bond. This C3 turnover mechanism serves as a surveillance system. If it deposits on healthy self, it is inactivated. If it deposits on a microbe, it can be rapidly amplified. Thus, in the latter case, C3b sequentially engages two proteases, factor B (FB) and factor D (FD), and the stabilizing protein properdin (P). These interact to form an AP C3 convertase (C3bBbP) that cleaves C3 to C3b and C3a. This system, therefore, represents a powerful feedback loop for the generation of C3b (Fig. 3).

The classical pathway (CP) was discovered first in the late 1800s, hence its name. It is primarily triggered by antibodies (IgM and IgG, subclasses 1 and 3) binding to antigens. This initiates a cascade featuring multiple proteolytic cleavage steps beginning with the C1s component of C1 that cleaves C4 and C2. The newly generated C4b and C2a fragments then interact to form the C3 convertase, a proteolytic complex that activates C3. This convertase “converts” C3 into the C3a fragment (an inflammatory modulator) and the larger fragment, C3b, which serves as an opsonin and nidus for a feedback loop to generate more C3b. In an analogous process, the LP generates the same C3 convertase, but in this scheme, lectins substitute for antibodies in binding antigen-like sugar (mannose) residues, and mannose binding lectin-associated serine proteases (MASPs) replace C1r and C1s [14].

All three pathways merge at the step of the generation of a C3 convertase (Fig. 2). The C3b that deposits on pathogens serves both as a ligand for receptors in addition to being a central component of the feedback loop. Addition of a second C3b to a C3 convertase generates a C5 convertase. The latter cleaves C5 into a potent anaphylatoxin (C5a) and the larger C5b fragment. The C5b binds to C6 and C7 (without proteolysis) and this complex attaches to a membrane. Next, the C5b67 complex binds C8 followed by multiple C9s (~10–15) to form the MAC (C5b-9) (reviewed in [15]).

### CD46 and the “regulators of complement activation” gene cluster

Control of complement occurs in the fluid phase (plasma) and on self-tissue at each of the major steps in the pathways: initiation; amplification leading to C3 and then C5 cleavage; and, formation of C5b-C9.

Originally identified as a C3b- and C4b-binding protein of human peripheral blood cells, CD46 is expressed as a type-I transmembrane protein on nucleated cells [16–18]. The *MCP* gene is located in the regulators of complement activation (RCA) cluster at 1q3.2 [6, 19]. In addition to CD46, other proteins in this C3/C4-interacting family are CD35 (complement receptor one; CR1); CD21 (complement receptor 2, CR2); CD55 (decay-accelerating factor, DAF); C4b binding protein (C4BP); factor H (FH) and its family of proteins, factor H-like protein 1 (FHL-1), and factor H-related proteins 1–5 (FHR-1 to 5) [7, 20, 21].

The human RCA gene cluster spans a total of 21.45 cM on the long arm of chromosome 1 and includes more than 60 genes of which 15 are related to complement [21]. One group of genes is telomeric in a 900-kb DNA segment and the other is a centromeric 650-kb fragment. These are separated by a 14–59 cM segment that includes a number of genes unrelated to complement [21]. Complement regulatory genes share a common ancestral motif from which they arose by multiple gene duplication events [13, 19, 21].

CD46 and other members of the RCA are composed of a repeating unit that begins at the amino-terminus and comprises most or all of the protein. This structural feature, called a complement control protein (CCP) module (also a short consensus repeat or a sushi domain), consists of ~60 amino acids with four invariant cysteines and 10–18 other highly conserved amino acids. The CCP modules house the sites for regulatory activities, i.e., binding sites for C3b, C4b and/or factor I (FI) that foster cofactor activity (CD46, FH, C4BP, CR1), and decay-accelerating activity (CD55, FH, C4BP, CR1).

**Fig. 4:**
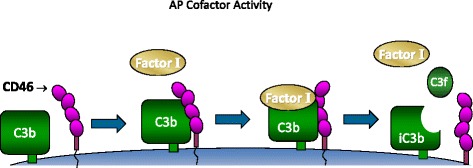
Cofactor activity of CD46 illustrated for the alternative pathway. CD46 binds to C3b that becomes attached to host cells. This then allows the serine protease factor I to cleave C3b into iC3b that cannot participate in the feedback loop. CD46 is nearly ubiquitously expressed on human cells

Cofactor activity is the process whereby C3b and C4b are proteolytically inactivated by FI, a plasma serine protease. A cofactor protein such as CD46 or FH must bind to the substrate (C4b or C3b) before FI can cleave these two large fragments (Fig. 4). Decay-accelerating activity (DAA) refers to a regulatory process for enhancing the spontaneous decay of the convertases. The active protease fragment is dissociated. CD46 possesses cofactor activity for C4b and C3b but does *not* possess DAA.

The gene for CD46 is ~43 kb and contains 14 exons [6]. A partial duplicate (exons 1–4; *MCP-like*) has been identified, but there is no evidence for its expression. CD46 consists of four major alternatively spliced transcripts [6, 16]. Most cell types express all four of the proteins in approximately the same ratio as is observed on peripheral blood cells (although cell-specific isoform expression has been identified [16, 19]). Each isoform shares an identical amino-terminal portion consisting of four CCPs followed by an alternatively spliced region for *O*-glycosylation. Although there are three exons coding for this region (A, B, and C), the four most commonly expressed isoforms carry B + C or C alone. Next is the flanking juxtamembraneous 12 amino-acid domain of unknown significance followed by a hydrophobic transmembrane region and a charged cytoplasmic anchor. The carboxyl-terminal tail is also alternatively spliced. It contains one of two nonhomologous cytoplasmic tails of 16 or 23 amino acids each of which houses signaling motifs. The four major isoforms are named CD46-BC1,−BC2,−C1, and − C2.

### CD46 function

In addition to its primary role in the regulation of C3b and C4b as a membrane cofactor protein, CD46 has been called the “multitasker” [18] and a “pathogen magnet” [22]. CD46 is increasingly recognized for its roles in linking innate and adaptive immune responses [17, 18, 23].

#### Pathogen magnet

The nearly universal expression of CD46 may particularly explain its exploitation by a number of pathogens who employ it as a receptor, possibly to co-opt one or more of its complement regulatory activities, signaling capabilities, or internalization mechanisms (reviewed in [22]). Nine human-specific pathogens target CD46 including *four* viruses (measles virus, adenovirus groups B and D, and herpesvirus 6) and *five* bacterial species (*Neisseria gonorrhoeae*, *Neisseria meningitidis*, *Streptococcus pyogenes*, *Escherichia coli*, and *Fusobacterium nucleatum*). Additionally, bovine CD46 is a receptor for bovine viral diarrhea virus, an enveloped RNA virus.

Pathogens target different domains of CD46 for attachment and subsequent reactions. The adenovirus fiber-knob protein and measles virus hemagglutinin attach to CCPs 1 and 2. Measles-virus binding elicits internalization and alters intracellular processing and antigen presentation (reviewed in [24, 25]). An envelope glycoprotein complex from herpesvirus 6 binds CCPs 2 and 3 (reviewed in [26]). Following clathrin-mediated endocytosis, the viral nucleic acid transits to the nucleus and replicates [25]. The type-IV pilus of the *Neisseria* species targets CCPs 3 and 4 and the STP segment. *Neisseria* infection causes the phosphorylation of the cytoplasmic domain (CYT-2) of CD46 by c-Yes, a member of the Src tyrosine kinase family. Studies suggest this phosphorylation is essential for *Neisseria* attachment and cytoskeletal rearrangement and that *Neisseria* also stimulates proteolytic cleavage of CD46 tails during infection (reviewed in [18, 27]). The M protein of *S. pyogenes*, which facilitates invasion of epithelial cells, attaches to CCPs 3 and 4. On epithelial cells, infection leads to the shedding of CD46 at the same time as the bacteria induce apoptosis and cell death [28]. Furthermore, interaction of *S. pyogenes* and CD46 triggers cell signaling pathways that lead to an immunosuppressive/regulatory phenotype in T cells (reviewed in [29] and [22]). The binding site for the periodontal disease-associated *F. nucleatum* is unknown [30]. However, the binding of *F. nucleatum* to CD46 on epithelial cells contributes to increasing levels of proinflammatory mediators and matrix metalloproteinases likely involved in periodontal tissue destruction [30].

Pathogenic microbes also develop their own human-like proteins to subvert host defenses. Poxviruses express CD46-like proteins (30–40 % homologous) to control host complement [31, 32]. Called poxviral inhibitors of complement enzymes (PICES), proteins from variola, and monkeypox are named SPICE (smallpox inhibitor of complement enzymes) and MOPICE (monkeypox inhibitor of complement enzymes), respectively [31–34]. They consist of three or four CCPs that are structural and functional mimics of CD46 and CD55. These virulence factors attach to glycosaminoglycans on the cell surface via their heparin-binding sites and, thus, downregulate the complement system’s ability to attack the virus [31].

Better understanding of the mechanism by which pathogens hijack (e.g., poxviruses) and usurp CD46 function may also provide greater insights relative to its normal functional repertoire. Additionally, replication-defective forms of adenovirus are being utilized in gene transfer and vaccine clinical trials necessitating a better understanding of its attachment mechanisms (reviewed in [25]).

#### Immunomodulatory functions

Over the last 15 years, it has become increasingly apparent that CD46 plays important and surprising immunomodulatory roles. These studies have been extensively reviewed particularly in relationship to T-cell biology (reviewed in [17, 18, 23]). The co-engagement of the T-cell receptor and CD46 by CD4^+^ T cells leads to the induction of interferon-γ (IFN-γ)-secreting effector T-helper type-1 (Th1) cells (reviewed in [17, 18, 23]). These subsequently switch predominantly into interleukin-10 (IL-10)-secreting regulatory T cells (Tregs). Thus, a time-ordered functionally-relevant sequence occurs following stimulation through CD46; activation initially induces a rapid burst of interleukin-2 (IL-2) secretion and the generation of a proinflammatory IFN-γ^+^/IL-10^−^T cell phenotype followed by an intermediate step with IFN-γ^+^/IL-10^+^ cells. The latter then switches to an IFN-γ^−^/IL-10^+^ self-regulatory phenotype (Treg). Recent studies suggest a possible mechanism for this via the binding by CD46 to a newly discovered extracellular ligand, Jagged1, a member of the Notch family of proteins that is regulated by CD46 [18].

Thus, one could envision that at the outset of an immune response, complement-induced Tregs provide a supportive role by facilitating B-cell activation via high IL-10 and sCD40L production (factors required for optimal B-cell activation and Ig class switching). As the immune response progresses, especially with antibody production and complement-mediated clearance of infectious pathogens, complement-induced Tregs might then constrain and/or deactivate the immune response.

Development of a Treg response is important for maintaining peripheral tolerance and control of immune responses. Further, T-cell subsets are increasingly recognized to possess different levels of plasticity in which they acquire new features and functions relative to secondary or chronic immune responses [35]. Thus, disruption or alterations of any of these pathways may contribute to susceptibility to infections or to the development of autoimmunity. Human diseases implicating defects in CD46-mediated signaling (reviewed in [17]) are multiple sclerosis, rheumatoid arthritis, asthma, IPEX-like syndrome, and primary C3 deficiency. Further, a subset of CD46-deficient patients develops common variable immunodeficiency (CVID), a syndrome characterized by hypogammaglobulinemia [36]. One study demonstrated that CD46-activated T cells support B-cell activation and that T cells from a CD46-deficient patient are impaired in promoting IgG production by B cells [37]. However, it is as yet unclear how these CD46-signaling functions play out in human disease.

### CD46 variants and disease association

Linkage analyses, genome-wide association studies, and the recent dramatic progress in next-generation sequencing have revealed an expanding number of disease-associated genetic alterations in CD46. Specifically, *MCP* variants have been increasingly associated with inflammatory disorders particularly characterized by the development of thrombi in small blood vessels (thombotic microangiopathy), especially atypical hemolytic uremic syndrome (aHUS) [38–43].

**Fig. 5:**
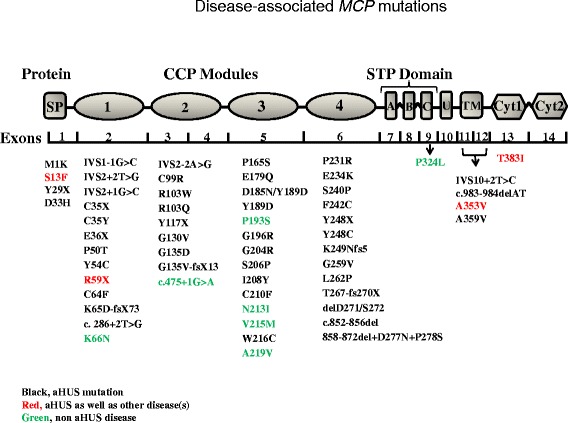
Disease-associated CD46 mutations. A schematic depicting CD46 protein, genomic organization, and disease-associated amino acid mutations. CD46 has a 34-amino-acid signal peptide (SP). The mature protein consists of four complement control protein (CCP) repeats that house the sites for regulatory activity. This is followed by an alternatively spliced region for *O*-glycosylation (segments A, B, C), a segment of undefined function (U), a transmembrane domain (TM), and one of two alternatively spliced cytoplasmic tails (CYT-1 or CYT-2). The gene consists of 14 exons and 13 introns for a minimum length of 43 kb. A majority of mutations for aHUS and for other disorders (such as systemic sclerosis, systemic lupus erythematosus, and pregnancy-related disorders) occur in the four CCPs. *Black*, aHUS mutations; *red*, aHUS and other diseases; *green*, non-aHUS disease

As expected, a majority of aHUS and other disease-associated mutations in CD46 occur in the four CCPs, the extracellular domains responsible for its complement regulatory activity (Fig. 5 and Tables 1 and 2). An earlier publication dissecting many of the active sites of CD46 by mutation modeling demonstrated how critical amino acids at binding sites lead to a loss-of-function [44].

**Table 1:** CD46 mutations associated with aHUS and functional consequences

Mutation^#^	Domain	Functional studies	Notes	Refs
M1K	SP	Reduced expression		[69]
S13F	SP	ND		[70]
Y29X	SP	ND	Compound heterozygote with factor H mutation	[43, 53]
D33H	SP	ND		[46]
IVS1 − 1G > C	CCP1	Reduced expression	Aberrant splicing of 2 bp after normal splice site; premature stop codon C35X	[36, 43, 47, 48]
IVS2 + 2 T > G	CCP1	Reduced expression	Deleted 144 bp and 48 amino acids in phase with wild-type	[36, 43, 47, 48, 71]
IVS2 + 1G > C	CCP1	Reduced expression		[39, 38, 71, 48, 43]
C35X	CCP1	Reduced expression		[43, 72]
C35Y	CCP1	Reduced expression		[43, 47, 48, 71]
E36X	CCP1	Reduced expression		[43, 71]
P50T	CCP1	ND		[43, 72]
Y54C	CCP1	ND	Successful treatment with eculizumab; transplanted	[73]
R59X	CCP1	Reduced expression		[43, 46, 47, 71, 74]
C64F	CCP1	Reduced expression	Varicella trigger; 16 y/o successfully treated with plasma exchange	[43, 75, 76]
K65D-fsX73	CCP1	ND		[43, 53]
c. 286 + 2 T > G	Between CCP1-2	ND	Frameshift leads to stop in CCP2	[45]
IVS2 − 2A > G	CCP2	Reduced expression	c. 287 − 2A > G; Lacks exon 3 and creates stop codon at L133X;	[36, 43, 45, 47, 48, 71, 77–79]
C99R	CCP2	Reduced expression		[43, 47, 48]
R103W	CCP2	Normal expression & C3/C4 regulatory activity		[38, 39, 43, 48, 61, 71]
R103Q	CCP2	ND	Compound heterozygote with factor H mutation	[43, 53]
Y117X	CCP2	Reduced expression		[46, 69]
G130V	CCP2	ND		[43, 53]
G135D	CCP2	Reduced expression		[69]
G135V-fsX13	CCP2	ND		[43, 74]
P165S	CCP3	Reduced expression		[38, 39, 43, 48, 53]
E179Q	CCP3	Normal to higher expression; 50 % loss regulatory activity		[36, 43, 48]
D185N/Y189D	CCP3	Reduced expression		[36, 43, 48]
Y189D	CCP3	Reduced expression		[43, 45, 48, 70–72, 80]
G196R	CCP3	Reduced expression; decreased C4b CA only (FI interaction site)		[36, 48, 53, 73, 81]
G204R	CCP3	ND		[43, 53]
S206P	CCP3	ND		[43, 53]
I208Y	CCP3	ND	Compound heterozygote with factor H mutation	[43, 53]
C210F	CCP3	ND	Compound heterozygote with factor-I mutation	[43, 53]
W216C	CCP3	ND	Near functional site per [44]	[43, 45]
P231R	CCP4	ND	Functional site per [44]	[43, 45]
E234K	CCP4	ND		[46]
S240P	CCP4	Normal expression; loss of C3b CA & binding	Originally numbered S206P	[43, 48, 51]
F242C	CCP4	Normal expression; reduced C3b/C4b binding and CA		[43, 45, 48, 53, 72]
Y248X	CCP4	Reduced expression		[43, 48, 52, 71, 72]
Y248C	CCP4	ND		[46]
K249N-fsX5	CCP4	ND		[46]
G259V	CCP4	Reduced expression; reduced C3b/C4b binding and CA	Compound heterozygote with FH mutations	[82]
L262P	CCP4	Reduced expression	aHUS pts successfully treated with eculizumab;	[83]
T267-fs270X	CCP4	Reduced expression	delA843-C844	[43, 48, 52, 72]
Del D271/S272	CCP4	Reduced expression	Originally numbered D237/S238	[43, 48, 51, 84]
c.852-856del	CCP4	Reduced expression	Originally numbered as 903-907del	[38, 39, 43, 48]
858-872del + D277N + P278S	CCP4	Reduced expression		[43, 47, 48]
IVS10 + 2 T > C	TM	Reduced expression	Exon 10 skipped changing aa 316–321 & adding a stop at 322	[43, 71, 74]
c.983-984delAT	TM	ND	Frameshift with stop	[43, 45]
A353V	TM	Normal expression and complement regulatory function	Uncommon variant; numerous studies and several disease implications; sometimes termed A304V	[43, 47, 48, 61]
A359V	TM	ND	Japanese pt; compound heterozygote with Y189D in CCP3	[70]
T383I	CYT-1	ND	Fatal infections triggered aHUS in 2 patients. Mother massive viral infection; son by *E. coli* O157:H7	[43, 53, 69]

^**#**^Numbered from SP and using ABC1 isoform or as noted for CYT-1. *Abbreviations*: *SP* signal peptide (34 amino acids), *CCP1-4* complement control protein modules 1–4, *UN* Segment of unknown function proximal to membrane (12 amino acids), *TM* transmembrane domain, *CYT-1* cytoplasmic tail 1 (16 amino acids), *CA* cofactor activity, *ND* not done

**Table 2:** CD46 mutations associated with aHUS and/or other diseases

Mutation^#^	Domain	Disease association	Functional studies	Notes	References
−366A > G	Promoter	Systemic sclerosis	Reduced expression	Polymorphic variant; rs2796268	[56]
−652A > G	Promoter	Systemic sclerosis	Reduced expression	Polymorphic variant; rs2796267	[56]
S13F	SP	HELLP; SLE; aHUS	ND		[57, 58, 70]
R59X	CCP1	aHUS and common variable immunodeficiency	Reduced expression	Homozygote	[36, 37]
K66N	CCP1	PE & SLE	Normal expression; reduced ability to regulate C4b	Dimerization site on structural model	[59]
c.475 + 1G > A	CCP2	TTP	Reduced expression	Splice-site single nucleotide variant; deletes G152-C157	[81]
P193S	CCP3 (indel)	Miscarriage	Normal expression & C3b/C4b regulatory activity		[60]
N213I	CCP3	Miscarriage	Reduced expression & C3b/C4b regulatory activity		[60]
V215M	CCP3	Glomerulonephritis	Expression normal	Patient also has A353V mutation	[62]
A219V	CCP3	SLE	ND		[57]
P324L	STP-C	Miscarriage	Reduced expression; Normal C3b/C4b regulatory activity		[60]
A353V	TM	Miscarriage; C3-glomerulonephritis; HELLP Syndrome; aHUS	Reduced complement control on cell surface		[47, 48, 60, 64]
T383I	CYT-1	Miscarriage; aHUS	Normal expression & C3b/C4b regulatory activity; but could disrupt phosphorylation site on tail		[60, 69]

*Abbreviations*: See Table 1 abbreviations, *SLE* systemic lupus erythematosus, *PE* preeclampsia, *TTP* thrombotic thrombocytopenic purpura, *HELLP* syndrome, hemolysis, elevated liver enzymes and low platelets

Note that there exists confusion in the literature detailing CD46-protein numbering of mutations since some references include the 34 amino acid signal peptide and all exons of the STP domain (for example, [38, 39, 45, 46]). Others do not include the signal peptide and/or may leave out the exon of the STP domain that is a rare protein product (for example, [36, 47, 48]). In this review, we have adopted the format of numbering from the translated protein, i.e., the signal peptide and including all exons of the protein as recommended by the Human Genome Variation Society.

Overall, two mutations have been identified in the promoter, four in the signal peptide, thirteen in CCP1, nine in CCP2, fourteen in CCP3, thirteen in CCP4, one in the STP region, four in the transmembrane domain, and one in cytoplasmic tail one (CYT-1) (Tables 1 and 2). While 52 mutations are associated with the development of aHUS, 13 may be associated with other diseases, and four mutations have been described in several diseases (see below).

#### Atypical hemolytic uremic syndrome

Mutation of CD46 has been linked most often to development of aHUS. The overall incidence of this rare disorder was estimated to be 2 in 1 million (10^6^) in a North American population (reviewed in [43]). Although aHUS is characterized by the triad of microangiopathic hemolytic anemia, thrombocytopenia (lowered platelet count) and acute renal failure, other organs such as brain, lung, and gastrointestinal tract can also be affected [38–43, 49] . Most typical HUS cases (~90 %) are epidemic in nature featuring diarrhea in association with an enteric infection from a verocytotoxin-secreting bacteria (e.g., *Escherichia coli* O157:H7). Following gastrointestinal infection, most patients recover, although 5–10 % will progress into enteropathic or Shigatoxin-producing *E. coli* (STEC) HUS, which has a good prognosis for recovery.

In contrast, atypical HUS (aHUS) is a more severe, non-diarrheal type that results from alternative pathway over-reaction on endothelial cells, particularly in the kidney. Penetrance is ~50 % with a relapsing and remitting course resulting in a post-mortality rate in the acute phase of ~25 %. For survivors, ~50 % will remain dialysis-dependent. Approximately 60 % of aHUS cases occur during childhood, and in a majority, the initial episode occurs before the age of 2 years (reviewed in [50]). In contrast to mutations in FH or FI, kidney transplantation for CD46-deficient individuals has a nearly normal success rate since the transplanted organ carries a normal level of CD46 expression [43, 46, 49].

Factors that are reported to precipitate aHUS include infections, pregnancy, trauma, or drugs. Why the kidney endothelium is the major site of organ damage is unknown. What is increasingly clear is that the fundamental defect in this disease is an inability to control the AP on damaged or stressed cell surfaces resulting in excessive and harmful activation on “altered self”. Dysfunction of mutated proteins can result from loss-of-function (in regulators responsible for cofactor activity) or gain-of-function (activating components, hyperactive C3 convertases). Both lead to inefficiently degraded C3b and abnormal persistence of C3 and C5 convertases that, in turn, generate excessive amounts of complement-pathway effectors. Further, C5b initiates the assembly of the MAC, leading to membrane injury, while C5a recruits and activates leukocytes and upregulates vascular adhesiveness. With the delicate balance between complement activation versus complement regulation perturbed on endothelial cells, the thrombotic microangiopathy ensues with vessel-wall thickening, cell engorgement, and destruction.

Mutations in *MCP* that predispose to aHUS were first identified in 2003 [51, 52]. *MCP* mutations were evaluated in three families [51] with a second group reporting a mutation in one family [52]. At present, at least 52 mutations linked to development of aHUS and 13 to other diseases have been reported (Tables 1 and 2). Mutations in *MCP* are found in ~10–20 % of aHUS patients. Most mutations are missense but nonsense, and splice-site variants are also observed (reviewed in [40, 43, 46, 48, 53–55]). The majority are also commonly rare, novel, and deleterious.

In about 75 % of cases, the mutant protein is not expressed. In the remainder, the aberrant protein is expressed but has a reduced or absent function, i.e., C3b- or C4b-binding and/or cofactor activity. Reduced cofactor activity for C3b impairs proper regulation of the AP. In addition to *MCP* mutations, a specific SNP haplotype block termed the *MCPggaac* haplotype in the *MCP* promoter region may be associated in vitro with reduced transcriptional activity (reviewed in [43]). This has been linked with an increased risk of aHUS but only in the setting of a causative variant in another AP component or regulator.

#### Other diseases

Mutations in *MCP* associated with diseases other than aHUS have been noted (Table 2). These studies involve a small number of patients and all will require confirmation by further investigations.

Systemic sclerosis is an autoimmune disease characterized by immune system activation, microvascular dysfunction, and tissue fibrosis. Scambi et al. reported an association between abnormally low CD46 expression in skin vessels in a subset of patients with two polymorphic variants (−366A > G, rs2796268 and −652A > G, rs2796267) in the *MCP* promoter [56]. These two SNPs have also been linked to enhanced severity of aHUS [38, 39].

Nonsynonymous *MCP* mutations (S13F and A219V) were implicated in earlier development of nephritis, but were not predisposing to systemic lupus erythematosus (SLE) or nephritis [57]. The A353V (rs353665573) is an uncommon polymorphism (1–5 %) that has been reported to be associated with aHUS and several other diseases discussed below. The S13F *MCP* mutation is also associated with development of the HELLP syndrome that features a combination of hemolysis, elevated liver enzymes, and low platelets [58]. This disorder occurs in about 0.5–0.9 % of all pregnancies and in 5–10 % of patients who develop severe preeclampsia [58].

Preeclampsia complicates 4–5 % of pregnancies worldwide, causing significant maternal and neonatal mortality. Pregnancy in women with systemic lupus erythematosus (SLE) and/or the antiphospholipid syndrome (APS) may be particularly susceptible to complement-mediated injury with increased risk of preeclampsia, placental insufficiency, retardation, fetal growth issues, and miscarriage. A study analyzing a SLE and/or APL Ab cohort (PROMISSE)-sequenced genes for complement *FI*, *FH*, and *MCP* and found heterozygous mutations in seven (18 %) [59]. Five had risk variants that had been previously identified in aHUS, and one had a novel mutation in CD46, K66N (identified as K32N in the paper since the 34 amino acid signal peptide was not counted) that impairs only regulation of C4b. The study suggested a linkage between excessive complement activation and disease pathogenesis in patients with SLE and/or APL Ab who develop preeclampsia.

Idiopathic, recurrent miscarriage has also been associated with mutations both in *MCP* and C4b-binding protein (*C4BP*) [60]. All exons coding for CD46, C4BP, and decay-accelerating factor (DAF; CD55) were sequenced in a cohort of 384 childless women with at least two miscarriages. In addition to the first-time identification of a disease association of *C4BP* mutation, four *MCP* variants were identified. One of the rare variants, P324L, had decreased expression, while N213I had both impaired expression and function. Two mutations that did not appear to affect complement regulatory function were located in the transmembrane domain, and a third one was in the cytoplasmic tail that could impair signaling function [6, 60, 61].

Of the ~ eight known aHUS cases of homozygous CD46 deficiency, three also developed common variable immunodeficiency [36, 37]. The remaining five patients all presented with subnormal IgG1 levels. T cells from CD46-deficient patients were not capable of promoting B-cell responses suggesting a defect in the ability to optimize B cell responses could account for this disease [37].

Mutation screening was also undertaken in 19 patients with C3 glomerulonephritis (a form of glomerulonephritis characterized by mesangial C3 deposits) [62]. One patient was a compound heterozygote for *MCP* mutations in both exon 5 (V215M in CCP3) and exon 11 (A353V in the transmembrane domain). The V215M (termed V181M in the paper) mutation occurred in a site determined to be functionally important in a previous investigation [44].

Studies in the last decade have demonstrated how dysregulation of the AP contributes to age-related macular degeneration (AMD) (reviewed in [63–67]). It is a leading worldwide cause of central vision loss in individuals over the age of 50. Hypomorphs (i.e., genetically-based changes resulting in functionally deficient complement inhibitors that control the alternative pathway) account for ~50 % of the attributable genetic risk for AMD. Although no mutations have yet been identified linking AMD and CD46, several studies suggest it may have a role (reviewed in [67]). CD46 is found in drusen, the hallmark of dry-type AMD. Lower expression is observed in the monocytes of patients with AMD [67]. Further, smoking has been directly linked to development of AMD, and cigarette smoke extract decreased CD46 expression in retinal epithelial cells [67]. A mouse model that knocked out CD46 (*Cd46*^*−/−*^) found increased levels of membrane-attack complex, and vascular endothelial growth factors were increased in the retina and choroid of mice deficient in CD46 [67]. Further, these mice also developed more severe retinal damage in a laser induced model of AMD [67].

## Conclusion

Rare and uncommon mutations in *MCP* lead to development of aHUS and possibly several other diseases such as pregnancy-related disorders and SLE. The pathophysiological implications of the defective functioning of CD46 in aHUS relate to an inability to sufficiently control the alternative pathway of complement. Incomplete penetrance of mutations is 50 %, indicating that additional genetic or environmental triggers are involved. For aHUS, the outcome for renal transplantation with a normal CD46 kidney is much more favorable than for other mutations in complement proteins (reviewed in [43, 47]). Additionally, the availability of a new therapeutic option for aHUS, the treatment with a mAb to C5 (eculizumab), induces a remission in most patients (reviewed in [49, 68]). With the advent of whole exome and whole genome sequencing reaching more reasonable costs, and in view of other putative conditions associated with CD46, additional disease associations are likely on the horizon.

## Abbreviations

aHUS: atypical hemolytic uremic syndrome
AP: alternative pathway of complement
AMD: age-related macular degeneration
CCP: complement control protein module
CR1: complement receptor one (CR1 CD35)
C4BP: C4b binding protein
DAA: decay-accelerating activity
DAF: decay-accelerating factor (CD55)
FH: factor H
FI: factor I
MAC: membrane-attack complex
MCP: membrane cofactor protein (CD46)
RCA: regulators of complement activation
STP: serine-threonine-proline-enriched domain of CD46
